# Zn(OAc)_2_-Catalyzing Ring-Opening Polymerization of *N*-Carboxyanhydrides for the Synthesis of Well-Defined Polypeptides

**DOI:** 10.3390/molecules23040760

**Published:** 2018-03-26

**Authors:** Yanzhao Nie, Xinmei Zhi, Haifeng Du, Jing Yang

**Affiliations:** 1 State Key Laboratory of Chemical Resource Engineering, Beijing Key Laboratory of Bioprocess, College of Life Science and Technology, Beijing University of Chemical Technology, Beijing 100029, China; nieyanzhao126@126.com (Y.N.); zxmlucky903@163.com (X.Z.); 2 Beijing National Laboratory for Molecular Sciences, CAS Key Laboratory of Molecular Recognition and Function, Institute of Chemistry, Chinese Academy of Sciences, Beijing 100190, China

**Keywords:** Lewis pair polymerization, polymerization catalysis, polymer synthesis, polypeptides, ring-opening polymerization

## Abstract

Despite notable progress, the fabrication of well-defined polypeptides via controlled ring-opening polymerization (ROP) of α-amino acid *N*-carboxyanhydrides (NCAs) using convenient catalysts under mild conditions in a relatively short polymerization time is still challenging. Herein, an easily obtained catalyst system composed of zinc acetate and aniline was explored to mediate the fast ROP of γ-benzyl-l-glutamate-*N*-carboxyanhydride (BLG-NCA) monomer, to produce poly(γ-benzyl-l-glutamates) (PBLGs) with controllable molecular weights and narrow dispersity. Considering the excellent cooperative action of zinc acetate and a broad scope of aniline derivatives with different functional groups to control ROP of BLG-NCA, this method may offer a useful platform enabling the rapid generation of end-functionalized PBLG and block copolymers for numerous biomedical applications.

## 1. Introduction

Polypeptides, as an important class of biopolymers, have found extensive applications in diverse biomedical areas, such as serving as scaffolds for tissue engineering, matrices for drug delivery, and responsive materials for biosensors [1,2,3,4,5]. The synthesis of polypeptides with well-defined structures has thus attracted the intense attention of chemists. The ring-opening polymerization (ROP) of α-amino acid *N*-carboxyanhydrides (NCAs) has witnessed a rapid growth and has become an important method for polypeptide synthesis since Deming and co-workers made a breakthrough by using transition metal complexes as active species [6,7]. Some organometallic (such as rare earth metal catalysts [8]) and metal-free initiators (e.g., hexamethyldisilazanes [9,10,11,12,13], tetrafluoroborate [14,15] and secondary amine-assisted primary amines [16,17,18,19]) or polymerization systems [20,21,22,23,24] were successively explored in the past decades. Despite these advances, some problems still await solution, such as the difficult access to initiators or catalysts, sensitivity of the catalysts to moisture and air, and long polymerization times. Therefore, it is highly desirable to develop simple and readily accessible catalysts to realize the controlled synthesis of polypeptides under mild conditions.

**Scheme 1:**
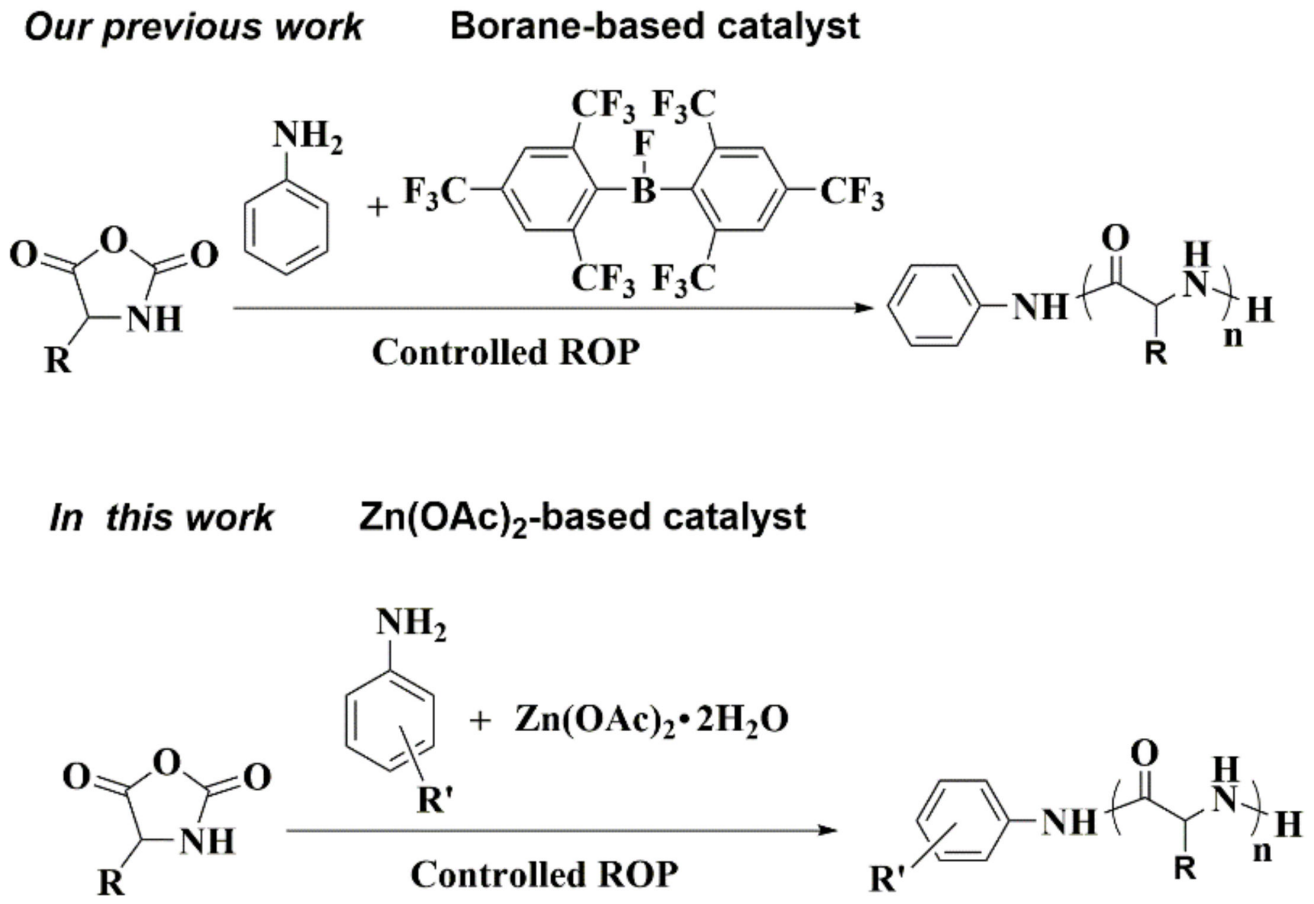
The reported boron-based strategy and the new approach proposed in this study. ROP- ring-opening polymerization.

The combination of Lewis acids (LAs) and bases (LBs) for cooperative activation of small molecules has attracted huge interest since the seminal work of Stephan [25,26,27,28,29,30,31,32]. In the past decade, this concept has shown remarkable versatility, and a wide range of Lewis pairs have been developed for the activation of small molecules, catalytic hydrogenation or hydrosilylation, and new reactivity development [33,34,35,36,37,38,39,40]. Recently, very promising results have also been reported in polymerization catalysis [41,42]. Indeed, Lewis pairs of aluminum-based Lewis acids and *N*-heterocyclic carbenes or phosphines have been explored in the polymerization of acrylic monomers [43,44,45,46,47,48,49]. Lactones have been precisely polymerized by highly interacting Lewis pairs for the synthesis of aliphatic polyesters [50,51,52,53,54]. As a continuation of our ongoing interest in Lewis pairs, we have recently reported a controlled ROP of NCAs using a frustrated Lewis pair of bulky bis-(2,4,6-tris-trifluoromethylphenyl)-fluoroborane (Fmes_2_BF) and aniline (Scheme 1) [55]. During the polymerization, the bulky Fmes_2_BF can capture the ω-amine end of a propagating chain to form a new Lewis pair intermediate, efficiently suppressing side reactions. Nevertheless, Fmes_2_BF lacks the ability to activate monomers, thus resulting in long polymerization times. Moreover, the complex preparation of boron derivatives for the synthesis of well-defined polypeptides might be also challenging.

Zinc alkoxides are known to efficiently propagate the ROP of cyclic esters and lactones [56,57,58]. Among a multitude of zinc complexes, zinc acetate (Zn(OAc)_2_) is readily available and stable under ambient conditions in the presence of moisture and air. However, to the best of our knowledge, the low-cost zinc acetate has seldom been reported to catalyze the polymerization of monomers. Aiming to develop simple and efficient catalytic polymerization systems, herein, the combination of zinc acetate and aniline/its analogues is explored for the controlled ROP of γ-benzyl-l-glutamate *N*-carboxyanhydrides (BLG-NCAs) (Scheme 1). Such a combination of zinc acetate and aniline exhibits exciting catalytic polymerization efficiency for BLG-NCA and produces well-defined poly(γ-benzyl-l-glutamates) (PBLGs) with controlled molecular weights and low dispersity in relatively short polymerization time. Moreover, zinc acetate tolerates well a broad spectrum of aniline analogues to promote the controlled ROP of BLG-NCA. In particular, combinations of zinc acetate and functionalized anilines derivatives offer an attractive approach for easily preparing PBLGs with special groups at the amino end of the polymer backbone to further satisfy the needs of numerous biological applications. For example, in the presence of zinc acetate, amantadine, an anti-influenza virus drug, can initiate BLG-NCA to afford well-defined drug-conjugated polypeptides. 

## 2. Results and Discussion

### 2.1. Controlled ROP of BLG-NCAMediated by Zn(OAc)_2_ and Aniline

Herein, aniline was selected as an example of such an initiator that contains a primary amine but has low nucleophilicity in comparison with the reported benzylamine or *n*-hexylamine. The polymerization of BLG-NCA initiated by aniline was firstly investigated in dichloromethane (DMC), *N*,*N*-dimethylformamide (DMF) and tetrahydrofuran (THF), respectively. Nearly 100% monomer conversion was achieved in 5.0 h in THF or DMF, and the obtained molecular weight (*M_n_*) was much higher than the calculated ones (runs 5 and 8 in Table 1). When the solvent was changed to DCM with a polar, non-coordinating solvent, the polymerization was rapidly completed in 1.0 h (run 2) with measured *M_n_* (1.35 × 10^4^), but with a molecular weight distribution (*Ð*) of 1.36. When Zn(OAc)_2_ and aniline cooperatively initiated BLG-NCA to polymerize in THF or DMF, the polymerization time was extended to 24 h (run 5 vs. run 6 in Table 1) and abroader *Ð* was obtained (run 8 vs. run 9). To our satisfaction, when the cooperative catalysis using Zn(OAc)_2_ and aniline in a molar ratio of 1 to 1 was performed in DCM, the polymerization proceeded completely in 3.0 h to afford PBLG with a dramatic decrease in *Ð* to 1.16 (Table 1, run 3).

**Figure 1:**
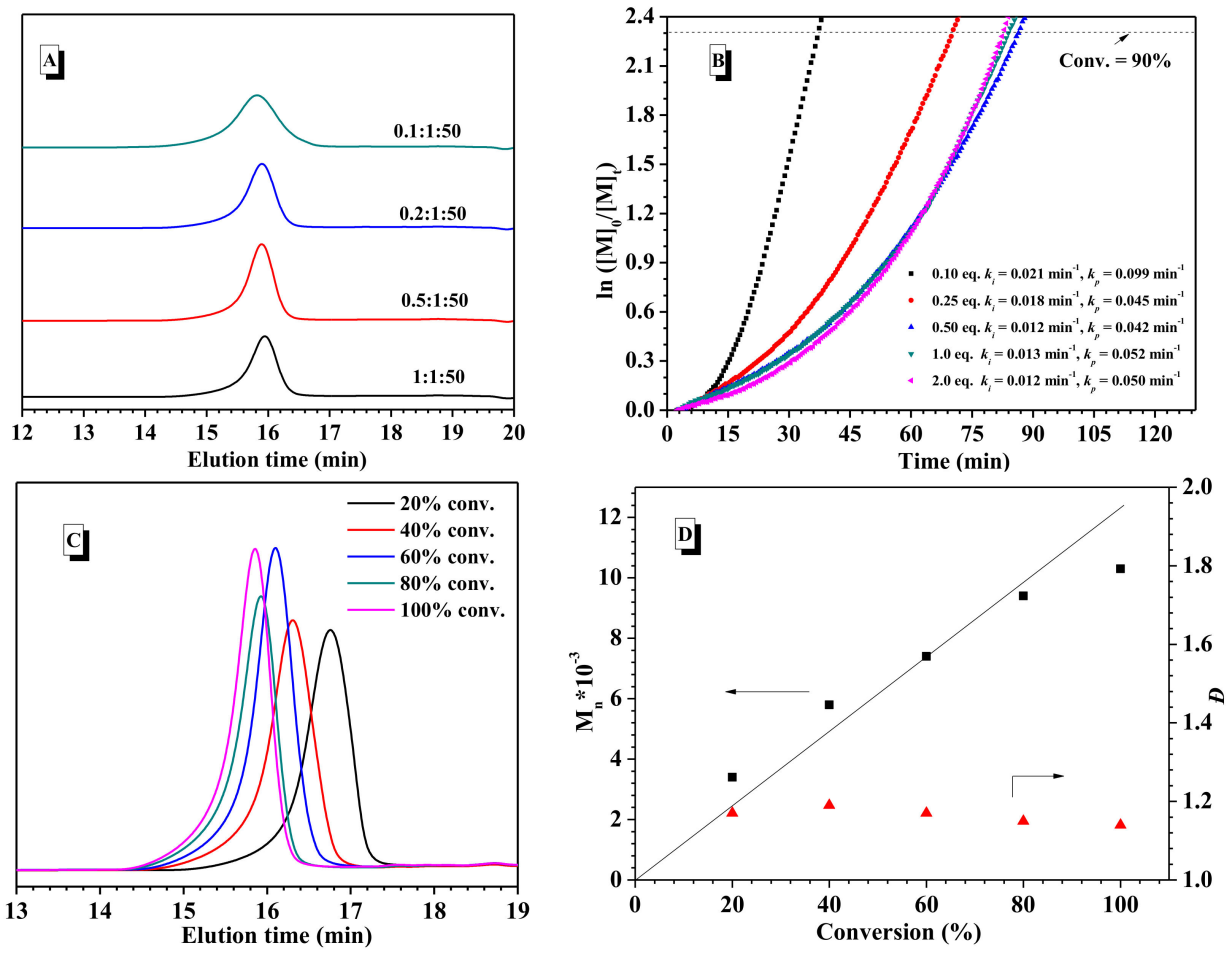
(**A**) GPC curves of poly(γ-benzyl-l-glutamates) (PBLG) synthesized with varying the molar ratio of Zn(OAc)_2_·2H_2_O and aniline. (**B**) Kinetics of the ring-opening polymerization (ROP) of γ-benzyl-L-glutamate-N-carboxyanhydride (BLG-NCA) initiated by Zn(OAc)_2_·2H_2_O and aniline system with varying molar ratio of Zn(OAc)_2_·2H_2_O equal. to aniline; (**C**) GPC profiles of PBLG depending on the different monomer conversion. (**D**) *M_n_* and *Ð* vs. BLG-NCA conversion. [BLG-NCA]/[Zn(OAc)_2_·2H_2_O]/[aniline] = 50/1/1, [BLG-NCA] = 0.75 M, at 25 °C in DCM.

Subsequently, several commercial available zinc salts were tested in this polymerization of BLG-NCA in DCM. Zinc chloride (ZnCl_2_) was found to inhibit the polymerization (Table 2, runs 1), and zinc trifluoromethane sulfonate (Zn(OTf)_2_) gave only 58% conversion of BLG-NCA in 20 h (Table 2, run 2). Interestingly, Zn(OAc)_2_·2H_2_O in place of Zn(OAc)_2_combined with aniline exhibited a similar result, but in a relatively shorter reaction time (Table 2, run 3). Under the same conditions, no polymerization occurred at all when Zn(OAc)_2_·2H_2_O was used alone. Therefore, the combination of Zn(OAc)_2_·2H_2_O and aniline was thus crucial to observe efficient and controlled polymerization of BLG-NCA. Due to the sparing solubility of Zn(OAc)_2_·2H_2_O in DCM, some suspended material was observed during the polymerization. The loading amount of Zn(OAc)_2_·2H_2_O was therefore investigated. As shown in Table 2, a decrease in the molar ratio of Zn(OAc)_2_·2H_2_O and aniline from 2:1 to 0.25:1 still gave good control over the polymerization (runs 5–7), and the polymerization time of BLG-NCA was only slightly shortened from 2.5 h to 2.0 h with 99% monomer conversion. When the ratio of Zn(OAc)_2_·2H_2_O and aniline was set to 0.1:1, at which some insoluble Zn(OAc)_2_·2H_2_O could be still observed, the polymerization went rapidly, but resulted in a relatively broad molecular weight distribution (run 4 in Table 2 and Figure 1A).

Kinetic studies were conducted for the polymerization of BLG-NCA with varying ratios of Zn(OAc)_2_·2H_2_O and aniline, to investigate the effects of the amount of Zn(OAc)_2_·2H_2_O on chain initiation and propagation. The progress of the polymerization was monitored in situ by IR at fixed time intervals (0.5 min), and a plot of ln([M]_0_/[M]_t_) versus time with gradually increasing slopes was observed (Figure 1B). Two stages were noted in the polymerization process: the early stage of the reaction with low initiation rate constant (*k_i_*) reflected from the slope and the propagation stage toward the end of the reaction with high propagation rate constant (*k_p_*). This was possibly associated with the lower nucleophilicity of aniline as initiator in comparison with the propagating species in the presence of Zn(OAc)_2_·2H_2_O. With an increase in the molar amount of Zn(OAc)_2_·2H_2_O from 0.1 to 0.5 relative to aniline, the initiation rate and the propagation rate were correspondingly decreased, whereas, upon further enhancing the amount of Zn(OAc)_2_·2H_2_O in the range between 0.5 and 2.0, similar polymerization kinetics were detected, which was attributed to the poor solubility of Zn(OAc)_2_·2H_2_O in DCM. 

The feeding ratios of BLG-NCA and the combination of Zn(OAc)_2_·2H_2_O and aniline were studied under the optimal condition (25 °C, 1.0 mL DCM, [BLG-NCA] = 0.75 M and [Zn(OAc)_2_·2H_2_O]/[aniline] = 1/1), as well. As the amount of BLG increased from 25 to 200, the polymerization could proceed completely to furnish the corresponding PBLG with the enhanced molecular weight and narrow distributions (Table 2, runs 8–12). Indeed, the polymerization could be carried out with two sequential additions of BLG-NCA (25/25 and 50/50) without any chain breaking reactions (runs 13 and 14 in Table 2 and Figure S2), and the obtained molar mass of the resultant PBLG were identical to those from the same amount of BLG in one batch addition. When BLG was polymerized in the presence of Zn(OAc)_2_·2H_2_O and aniline with a ratio of 1:1:50, the molar mass of PBLG versus time was further monitored by GPC measurements. As a result, it was found that *M_n_* of the obtained PBLG increased linearly with the reaction time (Figure 1C,D), and the low *Ð* was maintained in the range of 1.16–1.18. This indicated that the a combination of Zn(OAc)_2_·2H_2_O and aniline exhibited an excellent control over BLG-NCA polymerization under mild conditions in relatively short polymerization time.

**Figure 2:**
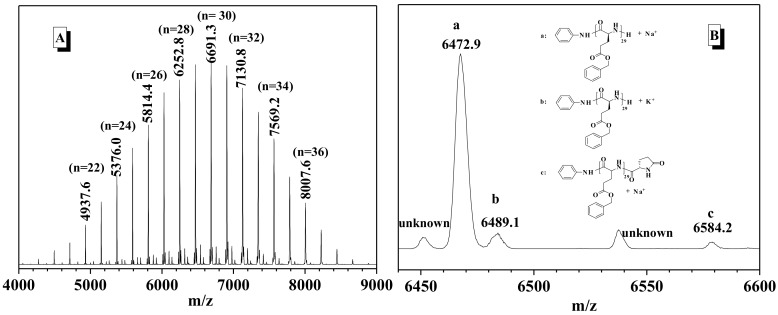
MALDI-TOF mass spectrum (**A**) and its enlarged region (**B**) of the poly(γ-benzyl-l-glutamates) (PBLG) accompanied by the proposed PBLG structures and their measured masses ionized with sodium or potassium ion.

The structure of the obtained PBLG was carefully analyzed by ^1^H-NMR (Figure S3). Because benzyl and amide signals from the repeating units of PBLG appeared in the range of 7.20 and 8.50 ppm, it was difficult to explicitly discriminate the presence of aniline at the chain end of the resultant PBLG. Thus, the polymer afforded at the feeding ratio of Zn(OAc)_2_·2H_2_O, aniline and BLG-NCA as 1:1:25 was further analyzed by MALDI-TOF mass spectrometry. As shown in Figure 2A, a series of peaks at 219*n* + 92 + 1 + 23 (*n* denotes the number of the repeating units) were assigned to be *n*(BLG-NCA–CO_2_) + C_6_H_5_NH + H + Na^+^ (Figure 2A). Thus the major product of BLG-NCA polymerization was the desired polypeptide bearing aniline via the amide bond to one chain end and new generated amine group on the other chain end. Meanwhile, additional distributions of peaks of very low intensities, such as 6584.2 Da in Figure 2B, was determined to be PBLG terminated with a pyroglutamate unit, which was resulted from intramolecular cyclization via attack of the amine end-group on the benzyl ester group as reported [59].

We attempted to monitor the structural change of aniline and the polymerization process of BLG-NCA in the presence of Zn(OAc)_2_ by NMR, but failed, probably due to the poor solubility of Zn(OAc)_2_·2H_2_O in DCM. Although the exact polymerization mechanism remains unclear at present, the results suggest that Zn(OAc)_2_·2H_2_O and aniline acted cooperatively to better control the ROP of BLG-NCA substantially which indicated the presence of an interaction between Zn(OAc)_2_·2H_2_O and aniline at the initiation stage. Moreover, combined with the relatively short polymerization time, it can be speculated that Zn(OAc)_2_·2H_2_O also plays a vital role to activate the BLG-NCA monomer, promoting the overall polymerization rate. These two words were separated with space.

### 2.2. Available Scope of Aniline Analogues

**Scheme 2:**
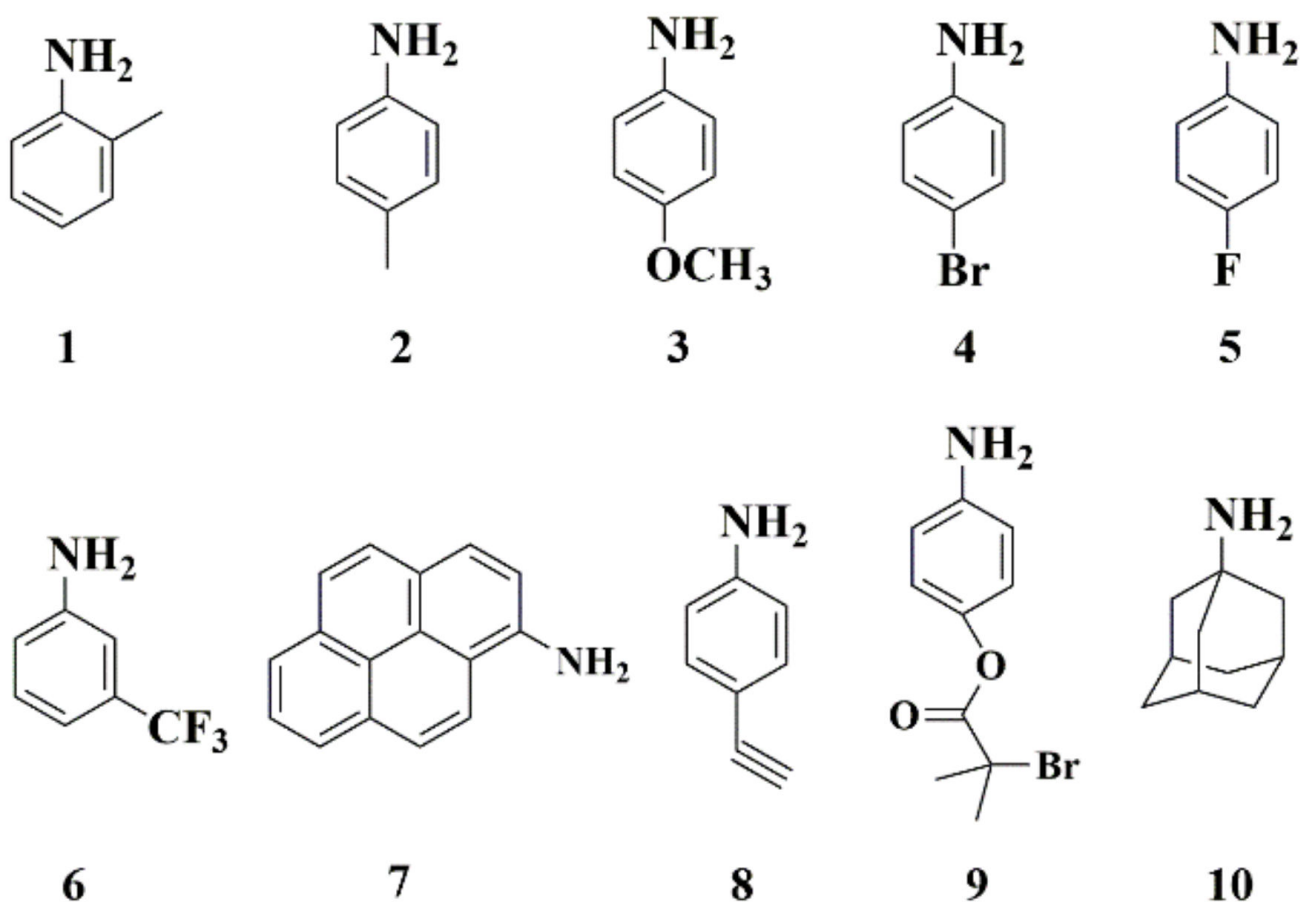
Chemical structures of aniline analogues investigated in this study.

Aiming at extending the scope of such ROP dual catalysis, our efforts were subsequently devoted to studying the tolerance of Zn(OAc)_2_·2H_2_O with aniline derivatives bearing either electron-donating or withdrawing substitutes (Scheme 2). All these polymerizations proceeded smoothly using combinations of Zn(OAc)_2_·2H_2_O and the corresponding amines, and exhibited good control over the polymerization of BLG-NCA (Table 3). Remarkably, Zn(OAc)_2_·2H_2_O and sterically congested anilines, such as Ana-1, were tolerated well in the polymerization, resulting in a narrow dispersity of 1.13 (run 1in Table 3). When Ana-2 and Ana-3 bearing electron-donating substituents were used in the presence of Zn(OAc)_2_·2H_2_O, PBLG with lower *Ð* was afforded (run 2 and 3 in Table 3) in comparison with the use of only the corresponding aniline derivatives. The aniline analogues 4~6 with electron-withdrawing groups were also suitable to combine with Zn(OAc)_2_·2H_2_O to efficiently control the ROP of BLG-NCA with a dramatic decrease in *Ð* from 1.63 to 1.29 (run 6 in Table S2 vs. run 6 in Table 3). In addition, the fluorescent 1-aminopyrene (Ana-7) was selected to cooperate with Zn(OAc)_2_·2H_2_O for BLG-NCA polymerization, and the obtained *Ð* was clearly decreased from 1.38 (in the absence of Zn(OAc)_2_·2H_2_O, run 7 in Table S1 and Figure S10) to 1.18 (Table 3, run 7). Moreover, fluorescence peaks of the resultant PBLG were clearly detected at 385 nm and 405 nm and attributed to the characteristic signal of pyrene ring (Figure S13), indicating the aminopyrene group on the PBLG structure. Also, Zn(OAc)_2_·2H_2_O showed good tolerance for alkynyl-functionalized anilines (Ana-8) to promote the ROP of BLG-NCA with narrow dispersity (*Ð* = 1.26) (Table 3, run 8). Such versatile carbon terminus of the resulting PBLG provided a great opportunity to link with other functional molecules or block polymers through alkyne-thiol reaction or alkyne-azide click-chemistry.

### 2.3. Synthesis of Functional PBLG with Well-Defined Structure

**Figure 3:**
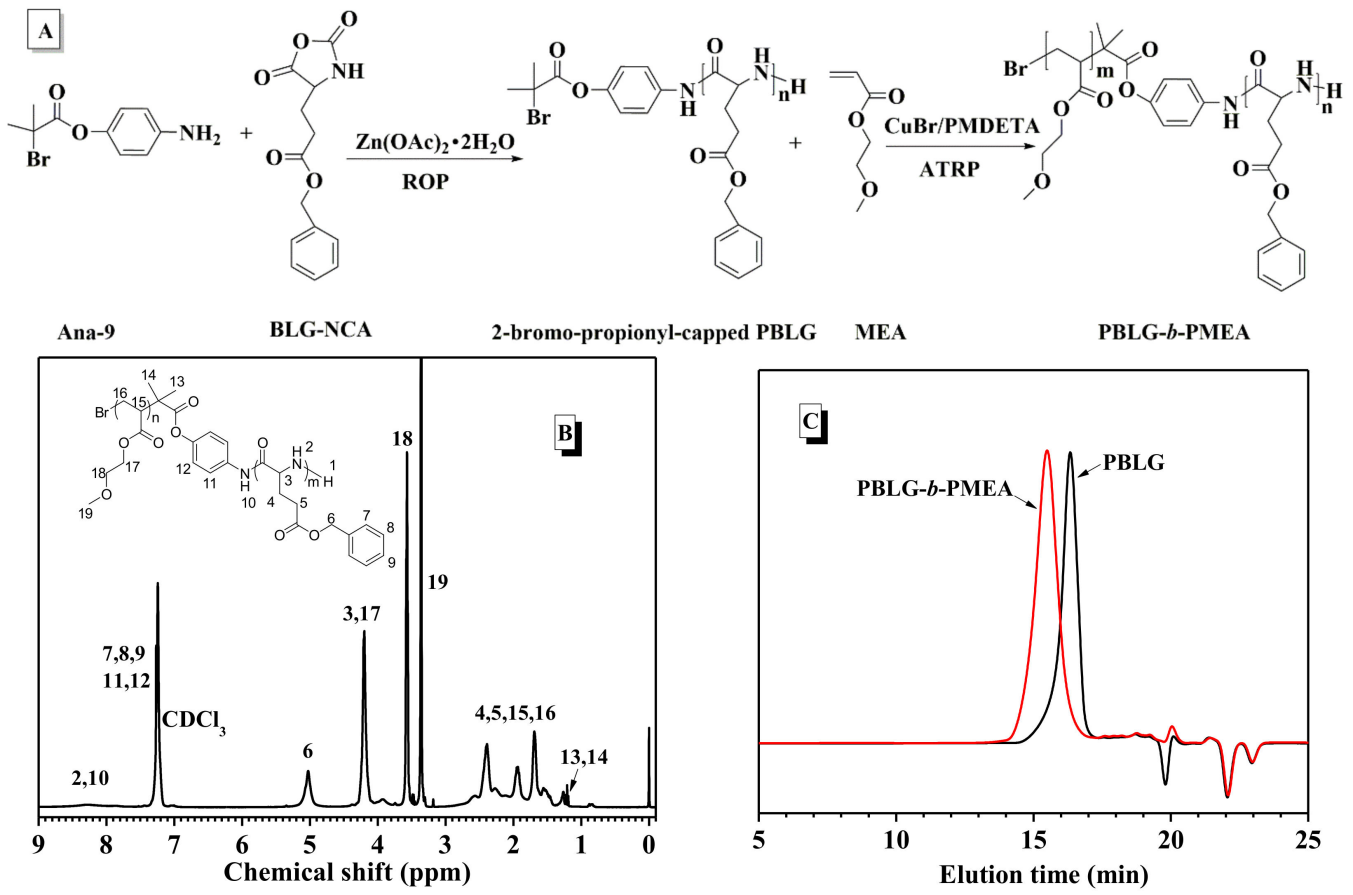
(**A**) Synthesis route of block polymer PBLG-*b*-PMEA; (**B**) ^1^H-NMR spectrum of PBLG-*b*-PMEA; (**C**) GPC curves of PBLG-*b*-PMEA.

Based on the preparation of aniline derivative Ana-9 containing 2-bromoisobutyryl group (Figure S14), the combination of Zn(OAc)_2_·2H_2_O and Ana-9 initiated the polymerization of BLG-NCA and exhibited satisfactory control over this polymerization, with a *M_n_* of 5600 and low dispersity (*Ð* = 1.23) (run 9 in Table 3). Subsequently, such 2-bromoisobutyryl-capped PBLG as macroinitiator further initiated MEA monomer to polymerize via atom transfer radical polymerization (ATRP, Figure 3A). The strong proton signals at 3.34 and 3.57 ppm (denoted as 18 and 19 in Figure 3B) associated with methyl and methylene around the ether group of the side chain indicated the presence of PMEA blocks in the final polymer. GPC measurements exhibited *M_n_* of 13,500 with unimodal and relatively narrow distribution (*Ð* = 1.30) without macroinitiator (Figure 3C), confirming the successful preparation of the block copolymer. 

**Figure 4:**
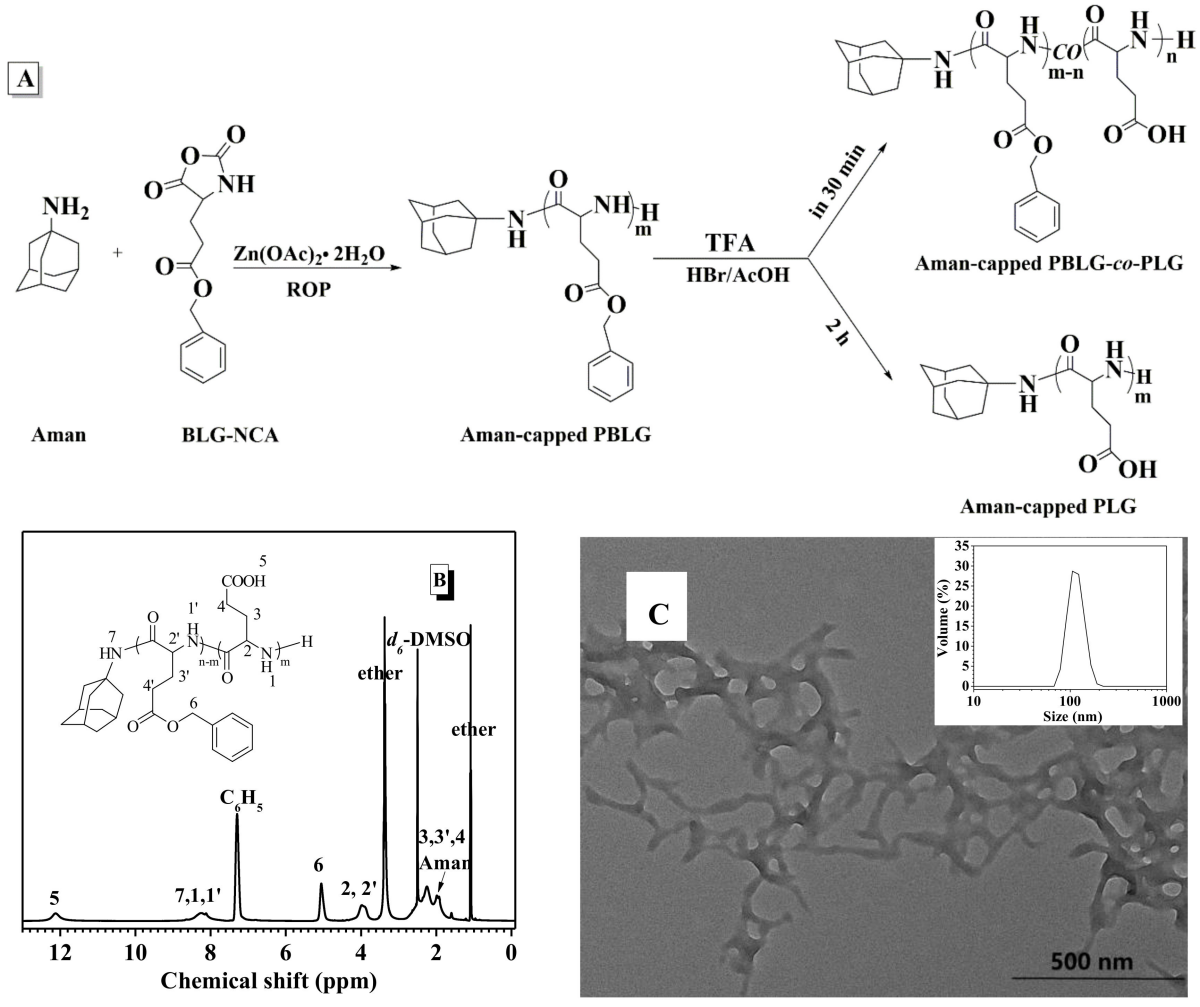
(**A**) Synthesis of Aman-conjugated poly(γ-benzyl-l-glutamates) (PBLG); (**B**) ^1^H-NMR spectrum of Aman-capped PBLG-*co*-PLG; (**C**) TEM image of the self-assembled morphology from Aman-capped PBLG-*co*-PLG with 50% benzyl groups content. Its DLS diagram is in the inset.

In fact, some drug molecules bearing amine groups could efficiently control the polymerization of BLG-NCA in the presence of Zn(OAc)_2_·2H_2_O, as well. For example, amantadine (Aman, Ana-10 in Scheme 2), an anti-influenza virus drug, was itself capable of initiating the polymerization of BLG-NCA, but an ill-defined polymer was obtained with broad dispersity (run 10 in Table S1 and Figure S15). Whereas, the polymerization was well controlled in the presence of Aman and Zn(OAc)_2_·2H_2_O, and the obtained *Ð* dramatically dropped from 1.48 to 1.13 (Table 3, run 10). The Aman-capped PBLG dissolved in TFA was further reacted with HBr (33 wt % in acetic acid) for 30 min to remove the benzyl protection groups (Figure 4A).The chemical structure of the resultant copolymer was confirmed by ^1^H-NMR measurements. As shown in Figure 4B, comparing the integral between the benzyl protons (denoted as 6, δ = 5.06 ppm) and the methine proton of the backbone (denoted as 2 and 2′, δ = 3.98 ppm), the afforded amphiphilic polymer Aman-capped PBLG-*co*-PLG had 50% remaining benzyl groups. In an aqueous environment, such a copolymer could self-assemble via hydrophobic forces to form Aman-containing nanocarriers (Figure 4C). The apparent average size was about 110 nm measured by DLS (inset in Figure 4C). Besides, when Aman-capped PBLG was incubated in the mixture of HBr and TFA for longer times, water-soluble Aman-conjugated PLG was successfully fabricated via the complete removal of the benzyl groups from the PBLG structure (Figure S16). This provided an efficient way to prepare water-soluble drug-conjugates with well-defined structure and enhance the solubility of a hydrophobic drug in a physiological environment. 

## 3. Materials and Methods 

### 3.1. Materials

Anhydrous DMC, DMF and THF purchased from J&K Scientific Ltd. (Beijing, China) were directly used without treatment. All aromatic amines except aniline were also purchased from J&K. Aniline was purchased from Sinopharm Chemical (Beijing, China). All liquid amines were dried with KOH for overnight, followedby distillation in vacuum at least three times. *p*-Anisidine and *p*-toluidine were recrystallized at least three times. H-Glu(OBn)-OH was purchased from GL Biochem Ltd. (Shanghai, China) and used as received. Amantadine (Aman) ordered from Alfa Aesar (Beijing, China) was used without further treatment. 4-Alkyl aniline purchased from J&K was recrystallized twice from dichloromethane. BLG-NCA was synthesized according to the reported procedure [60], followed by recrystallization four times prior to use. Zinc chloride (ZnCl_2_), zinc trifluoromethanesulfonate (Zn(OTf)_2_), zinc acetate and zinc acetate dihydrate (Zn(OAc)_2_·2H_2_O) purchased from Alfa Aesar were used without treatment. Trifluoroacetic acid (TFA) and HBr (33*wt*% in acetic acid) were ordered from J&K.2-Bromo-2-methylpropionic acid 4-ester aniline was prepared as report [61]. 2-Methoxyethyl acrylate (MEA) from Aldrich (Beijing, China) was distilled under vacuum and kept below 5 °C prior to use.

### 3.2. Characterization

The chemical structures of the monomer were characterized by ^1^H-NMR carried out on a 400 MHz NMR instrument (Bruker Corporation, Rheinstetten, Germany) at room temperature using CDCl_3_ as solvent. The chemical shifts were measured against the solvent signal of CDCl_3_ (δ = 7.26 ppm) as internal standard, respectively. GPC measurements of the synthetic polypeptides were carried out on an Agilent LC 1260 instrument (Agilent Technologies Inc., Calfornia, USA) equipped with a differential refractive-index detector. Aguard column and two 7.5 × 300 mm PLgel MIXED-C columnswere used. The measurements were performed using THF (containing 0.02 M LiBr) as eluent (flow rate of 1.0 mL/min at 35 °C), and polystyrene standards were employed for calibration. Polymer solutions at the concentration of 4.0 mg/mL were injected at an injection volume of 80 μL. In situ IR study of NCA polymerization was carried out by using a ReactIR^TM^ 15 equipped with a MCT Detector from Mettler Toledo AutoChem (Zurich, Swiss). The DiComp (diamond) probe was connected via AgX 6 mm × 2 m fiber (silver halide). Spectra were taken from 2000 to 650 cm^−1^ at 4 wavenumber resolution and the automatic sampling interval was 0.5 min. The calibrated curves for calculating the conversion of BLG-NCA during polymerization were plotted by the intensity ratio of the peaks at 1785 cm^−1^ (anhydride of monomer) to that at 1731 cm^−1^ (benzyl carbonyl group), based on preparing the mixture solution of monomer BLG-NCA and polymer PBLG with the different molar ratio. In the process of polymerization, the polymerization solution was taken out from the system, and spotted on the KBr plate for scanning by FT-IR (Nicolet Inc., Madisen, WI, USA). The accumulation rate was 16 times with 4 wavenumber resolution). The obtained intensity ratio at 1785 and 1731 cm^−1^ was compared with the calibrated curves (Figure S1 in Supplementary Materials) to afford the monomer conversion. Transmission electron microscopy (TEM) was performed on a HT7700 instrument (HitachiHigh-Technologies Corporation, Tokyo Japan) with 100 KV. Dynamic laser scattering (DLS) were measured at 660 nm by using a laser light scattering spectrometer (ZEN3600, Malvern Inc., Malvern, Britain) equipped with Zetasizer software. Desorption/ionization-time of flight mass spectrometer (MALDI-TOF MS) experiments were carried out on a Bruker Autoflex III (laser frequency 100 Hz, 355 nm and detector voltage of 1689 V). A Hitachi F-4600 Fluorescence instrument was used to measure the steady-state fluorescence emission of pyrene-capped PBLG.

### 3.3. Typical Polymerization Procedure

All polymerizations were carried out in a Vigor glovebox with a nitrogen atmosphere. The given amount of Lewis acid and aniline was stirred in 1.0 mL anhydrous CH_2_Cl_2_ for 10 min, followed by adding 197.4 mg BLG-NCA (0.75 mmol). The mixture was kept stirring at 25 °C, and a trace amount of the reaction mixture was taken out from the system at a predetermined time for monitoring the monomer conversion by IR spectroscopy. The final reaction solution was precipitated in anhydrous methanol, and washed with anhydrous methanol twice. The obtained polymers were dried in vacuum. 

### 3.4. Synthesis of Diblock Polymer PBLG-Block-PMEA 

A Schlenk flask equipped with a magnetic stir bar was charged with MEA monomer (195.2 mg, 1.5 mmol), PBLG macroinitiator (109 mg, 0.02 mmol), PMDETA (4.2 mg, 0.024 mmol), CuBr (2.9 mg, 0.02 mmol) and 1.0 mL dry and degassed dioxane. The flask was further degassed through three freeze-pump-thaw cycles. After the reaction system was immersed in an oil bath at 80 °C for 24 h, the polymerization solution was diluted with CHCl_3_, then purified through a neutral alumina column to remove copper. The concentrated reaction solution was dropwise added into cold diethyl ether, and the obtained polymer was dried in vacuum for 12 h. 

### 3.5. Preparation of Aman-Conjugated Poly(γ-benzyl-l-glutamate)-co-poly(l-glutamic acid) (Aman-Conjugated PBLG-co-PLG)

In a round bottom flask with a glass stopper and a stirrer bar, Aman-conjugated PBLG (200 mg) dissolved in 1.0 mL TFA was mixed with 2 equiv. of HBr (33 wt % in acetic acid) relative to per carbonyl group for 30 min. Then, the solution was poured into a large excess of cold diethyl ether, and a white precipitate was afforded in a yield of75%. ^1^H-NMR (DMSO-*d*_6_, δ ppm): 7.61–8.80 (N*H*CO), 7.01–7.42 (Ph-*H*), 4.80–5.20 (Ph-C*H*_2_), 3.60–4.42 (OCC*H*NH), 1.80–2.90 (Aman and C*H*_2_). Aman-conjugated poly(l-glutamic acid) (Aman-conjugated PLG) was obtained through a similar procedure except that the reaction time was extended to 6 h. ^1^H-NMR (DMSO-*d*_6_, δ ppm): 7.61–8.80 (N*H*CO), 3.60–4.42 (OCC*H*NH), 1.80–2.90 (Aman and C*H*_2_).

### 3.6. Preparation of Nanoparticles from Amphiphilic Copolymer Aman-Conjugated PBLG-co-PLG

The preparation of nanoparticles was carried out by slowly adding 0.5 mL DMF containing 5.0 mg Aman-conjugated PBLG-*co*-PLG into 10.0 mL ultrapure water. The nanoparticles solution was dialyzed against ultrapure water in a dialysis bag with a molecular weight cutoff of 3500 for 48 h, during which dialysis water was refreshed every 6 h. The concentration of the resulting nanoparticles was fixed at 0.5 mg/mL.

## 4. Conclusions

In summary, a simple operation and efficient approach for the preparation of well-defined synthetic polypeptides was developed using a combination of Zn(OAc)_2_·2H_2_O and anilines. Significantly, the tolerance of Zn(OAc)_2_·2H_2_O to a wide range of amine initiators paved the way for the convenient synthesis of highly desirable polypeptides with important functional groups, which would meet the requirements of synthetic polypeptides as biomaterials. Future work in our laboratory will seek to study the polymerization mechanism. The properties of the ensuing copolymers and drug-conjugated polymers will also be investigated to gain better knowledge on the influence of their functionality.

## Figures and Tables

**Table 1:** Polymerization of BLG-NCA in different solvents ^a^.

Run	Solvent	Zn(OAc)_2_:Aniline:BLG-NCA ^b^	Time ^c^ (h)	Conv. ^d^	*M_n.cal_* × 10^−4 e^ (g/mol)	*M_n,mea_*× 10^−4 f^ (g/mol)	*Ð* ^f^
1	DCM	0:0:50	24.0	0	-	-	-
2	DCM	0:1:50	1.0	100%	1.10	1.35	1.36
3	DCM	1:1:50	3.0	100%	1.10	0.90	1.16
4	THF	0:0:50	24.0	0	-	-	-
5	THF	0:1:50	5.0	100%	1.10	3.50	1.35
6	THF	1:1:50	24.0	86%	0.95	1.64	1.31
7	DMF	0:0:50	24.0	0	-	-	-
8	DMF	0:1:50	5.0	100%	1.10	4.18	1.35
9	DMF	1:1:50	8.0	100%	1.10	1.57	1.70

^a^ Carried out at 25 °C. ^b^ Indicating that the feeding molar ratio of Zn(OAc)_2_, aniline and γ-benzyl-L-glutamate-N-carboxyanhydride (BLG-NCA). ^c^ Indicate polymerization time. ^d^ Determined by monitoring the change of NCA anhydride absorption peak at 1785 cm^-1^ in FT-IR spectroscopy, and polymerization time was contained in the bracket. ^e^ Calculated by [BLG-NCA]/[aniline] × (*M_NCA_*-44) × monomer conversion. ^f^ Determined by GPC, *Ð* represents molecular weight distribution.

**Table 2:** Polymerization of γ-benzyl-l-glutamate-*N*-carboxyanhydride (BLG-NCA) catalyzed by aniline and various zinc salts in DCM ^a^.

Run	Zinc Salts	Zinc Salts:Aniline:BLG-NCA ^b^	Time (h)	Conv.^c^	*M_n.cal_* × 10^−4 d^ (g/mol)	*M_n,mea_*× 10^−4 e^ (g/mol)	*Ð* ^e^
1	ZnCl_2_	1:1:50	20	0	0	—	—
2	Zn(OTf)_2_	1:1:50	20	58	0.64	0.23	1.27
3	Zn(OAc)_2_·2H_2_O	1:1:50	2.5	>99	1.10	0.90	1.16
4	Zn(OAc)_2_·2H_2_O	0.1:1.50	1.0	>99	1.10	0.95	1.36
5	Zn(OAc)_2_·2H_2_O	0.25:1:50	2.0	>99	1.10	0.95	1.17
6	Zn(OAc)_2_·2H_2_O	0.5:1:50	2.5	>99	1.10	0.95	1.15
7	Zn(OAc)_2_·2H_2_O	2:1:50	2.5	>99	1.10	0.95	1.16
8	Zn(OAc)_2_·2H_2_O	1:1:25	2.5	>99	0.56	0.45	1.16
9	Zn(OAc)_2_·2H_2_O	1:1:75	3.0	>99	1.65	1.76	1.16
10	Zn(OAc)_2_·2H_2_O	1:1:100	4.5	>99	2.20	2.59	1.13
11	Zn(OAc)_2_·2H_2_O	1:1:150	5.0	>99	3.30	2.76	1.14
12	Zn(OAc)_2_·2H_2_O	1:1:200	8.0	>99	4.40	4.24	1.12
13	Zn(OAc)_2_·2H_2_O	1:1:(25 + 25)^f^	2.0 + 2.0 ^g^	>99	1.10	0.98	1.18
14	Zn(OAc)_2_·2H_2_O	1:1:(50 + 50)^f^	2.0 + 3.0 ^g^	>99	2.20	2.48	1.18

^a^ Carried out at 25 °C. ^b^ Indicating that the feeding molar ratio of Zn(OAc)_2_·2H_2_O, aniline and γ-benzyl-L-glutamate-N-carboxyanhydride (BLG-NCA). ^c^ Determined by monitoring the change of NCA anhydride absorption peak at 1785 cm^−1^ in FT-IR spectroscopy. ^d^ Calculated by [BLG-NCA]/[aniline] × (*M_NCA_*-44) × monomer conversion. ^e^ Determined by GPC, *Ð* represents molecular weight distribution. ^f^ BLG-NCA monomer was added in the two sequences. ^g^ Polymerization time for complete conversion of monomer in each sequence.

**Table 3:** Polymerization results of γ-benzyl-l-glutamate-*N*-carboxyanhydride (BLG-NCA) catalyzed by a combination of Zn(OAc)_2_·2H_2_O and various aniline analogues in DCM *^a^.*

Run	Analogues	Time (h) *^b^*	*M_n,exp_*× 10^−4^ *^c^* (g/mol)	*M_n,mea_*× 10^−4^ *^d^* (g/mol)	*Ð* *^d^*
1	Ana-1	2.5	1.10	1.01	1.13
2	Ana-2	2.5	1.10	0.87	1.10
3	Ana-3	2.5	1.10	0.95	1.11
4	Ana-4	2.5	1.11	0.97	1.31
5	Ana-5	2.0	1.10	1.18	1.21
6	Ana-6	6.5	1.11	1.62	1.29
7 *^e^*	Ana-7	3.5	0.57	1.14	1.18
8	Ana-8	2.5	1.10	0.97	1.26
9 *^e^*	Ana-9	2.5	0.57	0.56	1.23
10	Ana-10	1.0	1.11	2.80	1.13

*^a^* Performed by molar ratio of Zn(OAc)_2_·2H_2_O, aniline analogues and BLG-NCA with 1:1:50 except run 7 and run 9 at 25 °C. *^b^* The polymerization time for 99%monomer conversion. *^c^* Calculated by ([aniline analogues]-1) + [BLG-NCA]/[aniline analogues] × (*M*_NCA_-44) × monomer conversion. *^d^* Determined byGPC, *Ð* represents molecular weight distribution. *^e^* [Zn(OAc)_2_·2H_2_O]/[aniline analogues]/[M] = 1/1/25.

## Supplementary Materials

The following are available online, Figure S1: Calibrated curve of BLG-NCA conversion vs the peak intensity ratio at 1785 cm^−1^ and 1731 cm^−1^; Figure S2. GPC curves of PBLG prepared in the sequential addition. (A) 25/25; (B) 50/50.; Figure S3. ^1^H-NMR spectrum of PBLG catalyzed by Lewis pair of Zn(OAc)_2_·2H_2_O and aniline; GPC profiles of PBLG initiate by Ana-1 (Figure S4), Ana-2 (Figure S5), Ana-3 (Figure S6), Ana-4 (Figure S7), Ana-5 (Figure S8), Ana-6 (Figure S9), Ana-7 (Figure S10), Ana-8 (Figure S11) and Ana-9 (Figure S12) with or without Zn(OAc)_2_·2H_2_O; Figure S13. Fluorescent spectrum of PBLG initiated by a combination of Zn(OAc)_2_·2H_2_O with 1-aminopyrene; Figure S14. ^1^H-NMR (A) and ^13^C NMR (B) spectra of 2-bromo-2-methylpropionic acid 4-nitrophenyl ester; Figure S15. GPC profiles of PBLG initiate by Ana-10 with or without Zn(OAc)_2_·2H_2_O; Figure S16. 1H-NMR spectrum of Aman-capped PLG; Table S1. Polymerization results of BLG-NCA catalyzed by various aniline analogues without Zn(OAc)_2_·2H_2_O in CH_2_Cl_2_; Scheme S1. Synthesis route of Ana-9.
