# Supplementary material for: Zn(OAc)2-Catalyzing Ring-Opening Polymerization of N-Carboxyanhydrides for the Synthesis of Well-Defined Polypeptides
Source: Molecules. 2018 Mar 26;23(4):760. doi: 10.3390/molecules23040760 (PMC6017970; doi:10.3390/molecules23040760)
Supplement: Supplementary file 1 [file molecules-23-00760-s001.pdf]

Supporting information

$Zn(OAc)_2$-Catalyzing Ring-Opening Polymerization of *N*-Carboxy-Anhydrides for Synthesis of Well-Defined Polypeptides

Yanzhao Nie,[1] Xinmei Zhi,[1] Haifeng Du[2,*] and Jing Yang[1,*]

[1]State Key Laboratory of Chemical Resource Engineering, Beijing Key Laboratory of Bioprocess, College of Life Science and Technology, Beijing University of Chemical Technology, Beijing 100029, China. Email: yangj@mail.buct.edu.cn

[2]Beijing National Laboratory for Molecular Sciences, CAS Key Laboratory of Molecular Recognition and Function, Institute of Chemistry, Chinese Academy of Sciences, Beijing 100190, China.

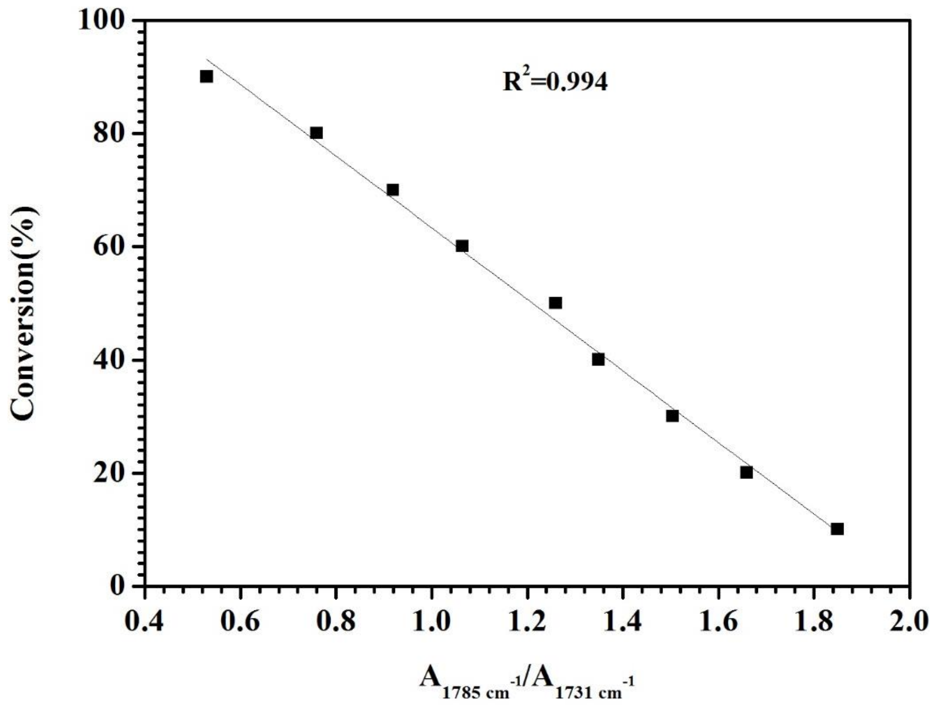

Figure S1. Calibrated curve of BLG-NCA conversion vs the peak intensity ratio at 1785 cm^{-1} and 1731 cm^{-1}.

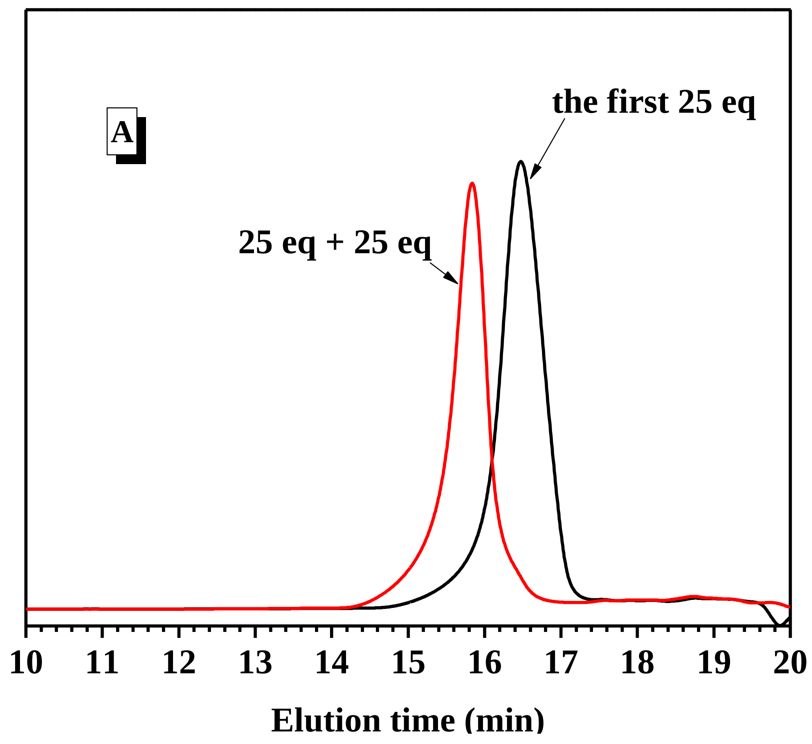

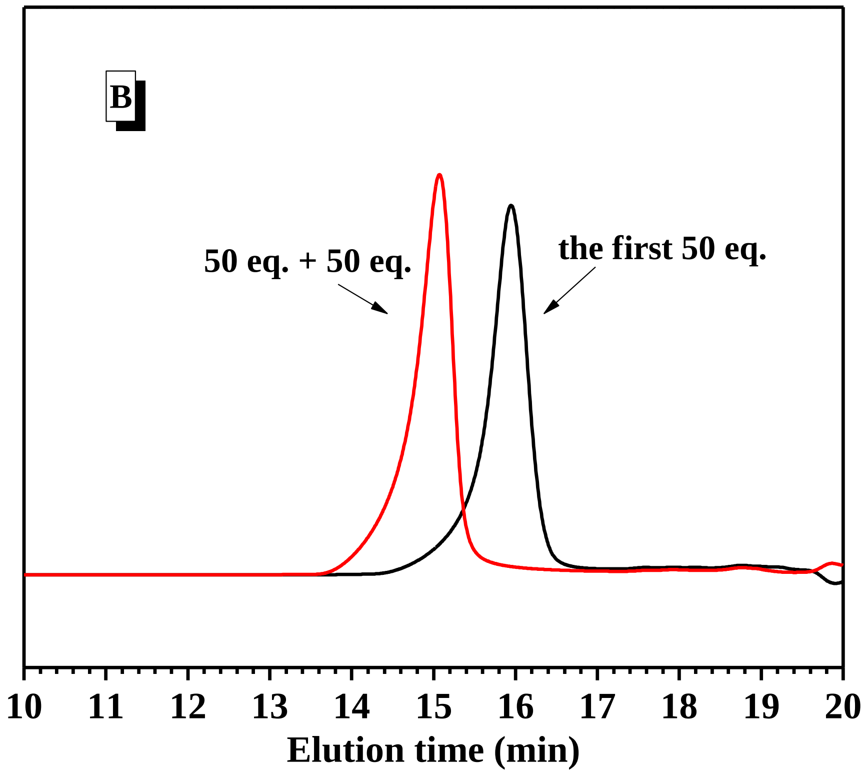

Figure S2. GPC curves of PBLG prepared in the sequential addition. (A) 25/25; (B) 50/50. [Zn(OAc)$_2$•2H_2O]/[aniline] = 1/1, [BLG-NCA] = 0.75 M, at 25 ºC in CH_2Cl_2.

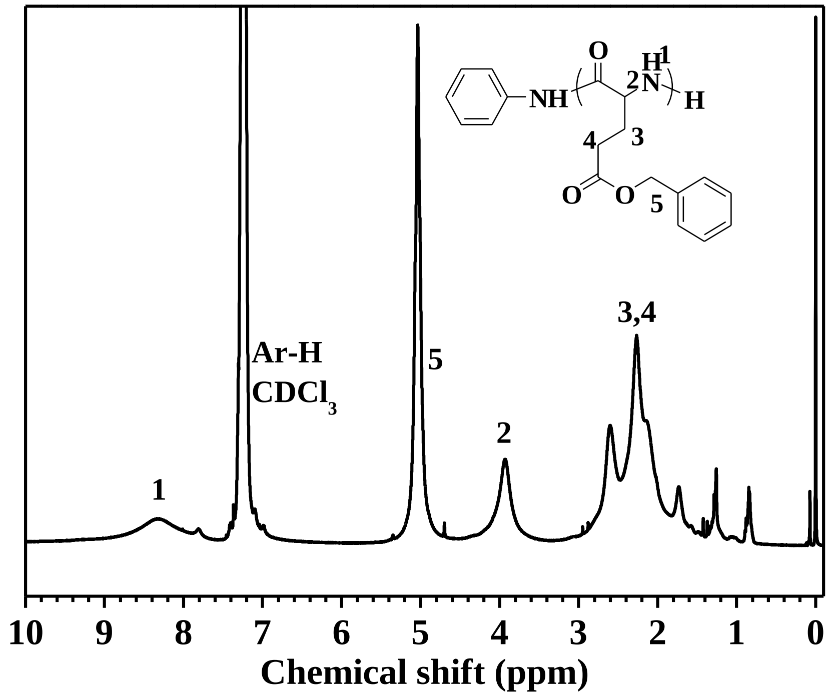

Figure S3. ^{1}H NMR spectrum of PBLG catalyzed by Lewis pair of Zn(OAc)$_2$•2H_2O and aniline

Table S1. Polymerization results of BLG-NCA catalyzed by various aniline analogues without $Zn(OAc)_2$•$2H_2O$ in CH_2Cl_2.[a]

Run	Ana	Ana:M	Time (h)[b]	$M_{n,cal}$×10^{-4} [c]	$M_{n,mea}$×10^{-4} [d]	Đ [d]
1	**Ana-1**	1:50	1.5	1.10	2.72	1.28
2	**Ana -2**	1:50	4.0	1.10	6.00	1.42
3	**Ana -3**	1:50	1.0	1.10	1.32	1.32
4	**Ana -4**	1:50	2.5	1.10	3.31	1.54
5	**Ana -5**	1:50	2.0	1.10	1.50	1.38
6	**Ana -6**	1:50	7.0	1.10	1.93	1.63
7	**Ana -7**	1:25	3.5	0.57	2.97	1.38
8	**Ana -8**	1:50	2.5	1.10	1.43	1.42
9	**Ana -9**	1:25	1.0	0.57	2.39	1.33
10	**Ana -10**	1:50	1.0	1.10	4.29	1.48

[a] Performed by at 25 °C. [b] The polymerization time for 99% monomer conversion. [c] Calculated by ([Ana]-1)+[BLG-NCA]/[Ana]×(M_{NCA}-44)×monomer conversion. [d] Determined by GPC, *Đ* represents molecular weight distribution.

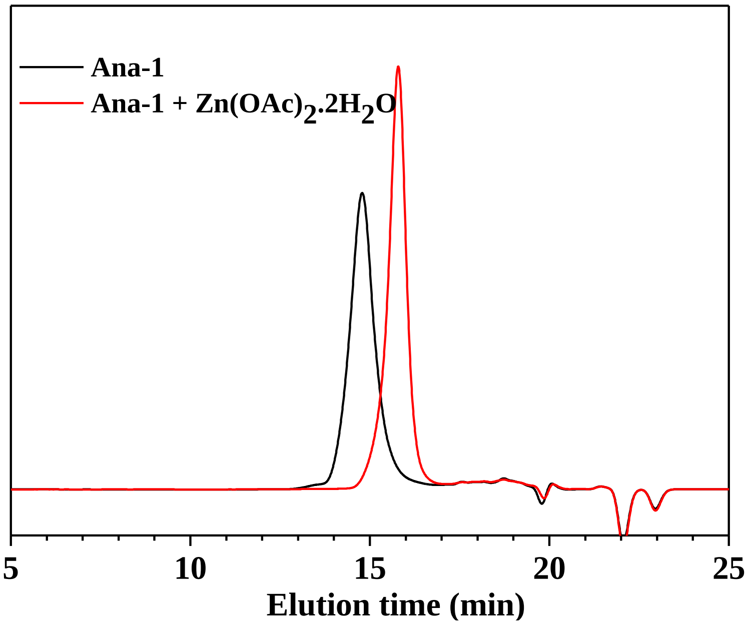

Figure S4. GPC profiles of PBLG initiate by Ana-**1** with or without $Zn(OAc)_2$•$2H_2O$. [BLG-NCA]/[$Zn(OAc)_2$•$2H_2O$]/[Ana -1] = 50/1/1, [BLG-NCA] = 0.75 M, at 25 °C in CH_2Cl_2.

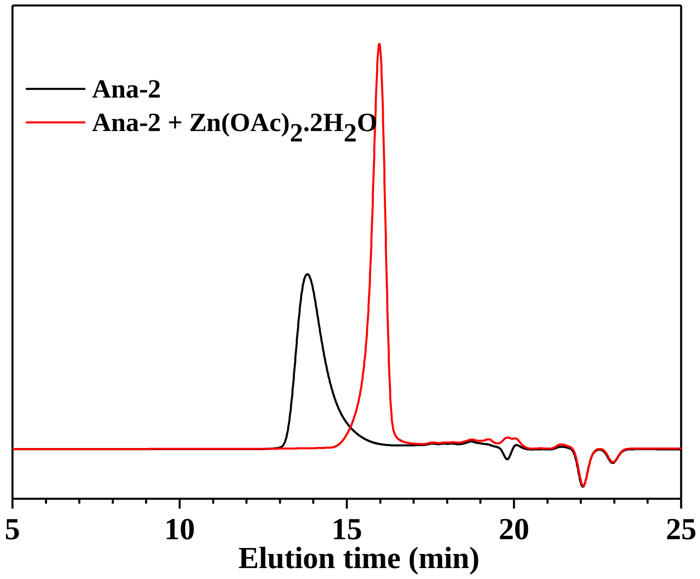

Figure S5. GPC profiles of PBLG initiate by Ana-**2** with or without $Zn(OAc)_2{\bullet}2H_2O$. [BLG-NCA]/[$Zn(OAc)_2{\bullet}2H_2O$]/[Ana-2] = 50/1/1, [BLG-NCA] = 0.75 M, at 25 ºC in CH_2Cl_2.

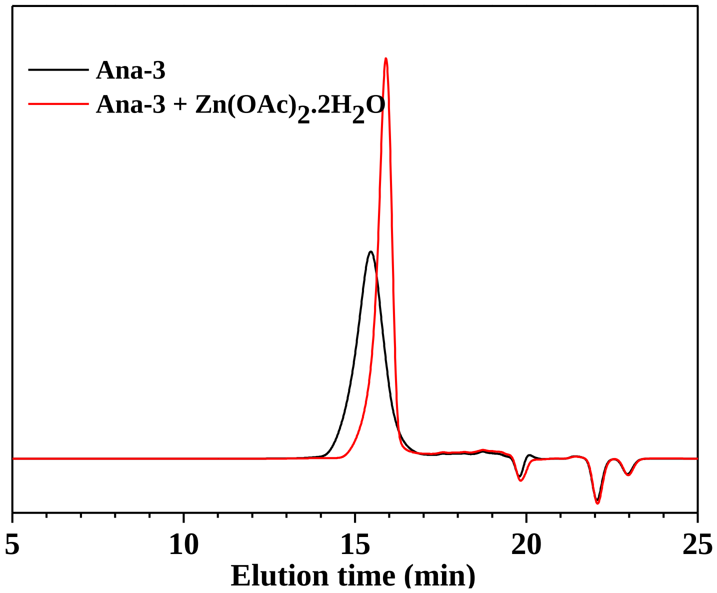

Figure S6. GPC profiles of PBLG initiate by Ana-**3** with or without $Zn(OAc)_2{\bullet}2H_2O$. [BLG-NCA]/[$Zn(OAc)_2{\bullet}2H_2O$]/[Ana-2] = 50/1/1, [BLG-NCA] = 0.75 M, at 25 ºC in CH_2Cl_2.

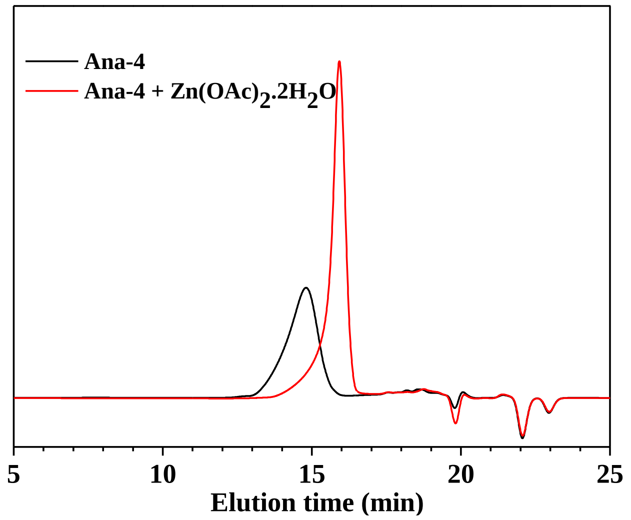

Figure S7. GPC profiles of PBLG initiate by Ana-**4** with or without $Zn(OAc)_2$•$2H_2O$. [BLG-NCA]/[$Zn(OAc)_2$•$2H_2O$]/[Ana-4] = 50/1/1, [BLG-NCA] = 0.75 M, at 25 ºC in CH_2Cl_2.

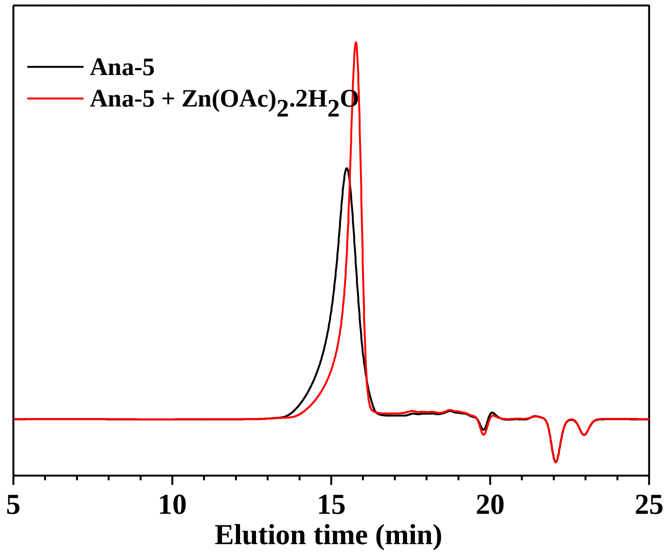

Figure S8. GPC profiles of PBLG initiate by Ana-**5** with or without $Zn(OAc)_2$•$2H_2O$. [BLG-NCA]/[$Zn(OAc)_2$•$2H_2O$]/[Ana-5] = 50/1/1, [BLG-NCA] = 0.75 M, at 25 ºC in CH_2Cl_2.

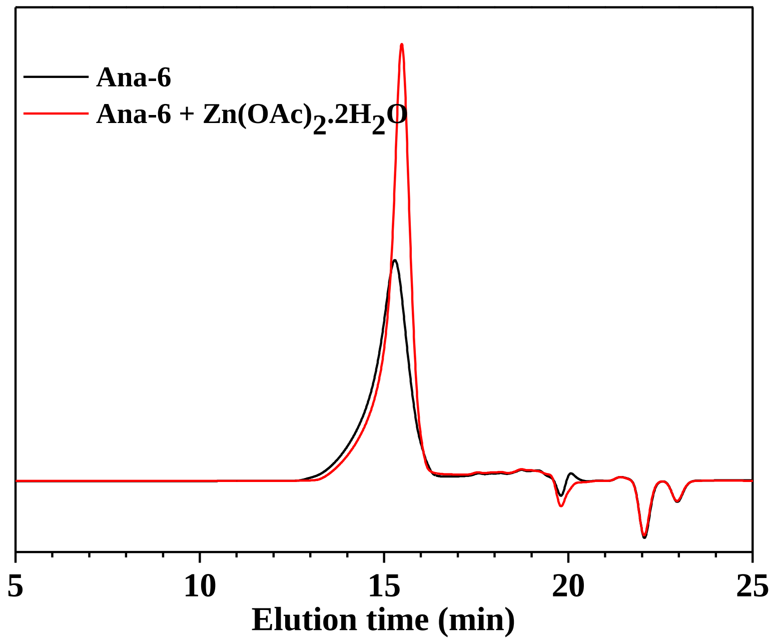

Figure S9. GPC profiles of PBLG initiate by Ana-**6** with or without $Zn(OAc)_2•2H_2O$. [BLG-NCA]/[$Zn(OAc)_2•2H_2O$]/[Ana-6] = 50/1/1, [BLG-NCA] = 0.75 M, at 25 ºC in CH_2Cl_2.

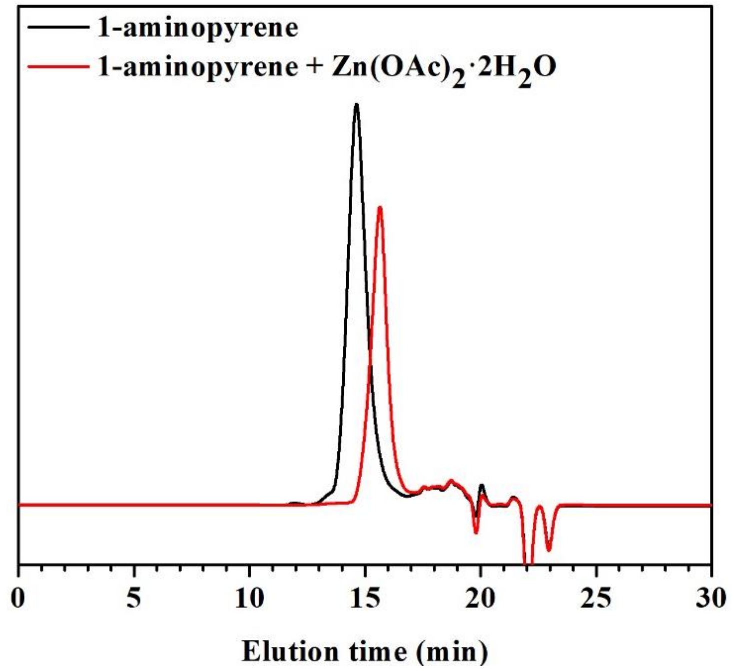

Figure S10. GPC curves of PBLG with Ana-**7** with or without $Zn(OAc)_2•2H_2O$. [$Zn(OAc)_2•2H_2O$]/[Ana-7]/[BLG-NCA] = 1/1/25, [BLG-NCA] = 0.75 M, at 25 ºC in CH_2Cl_2.

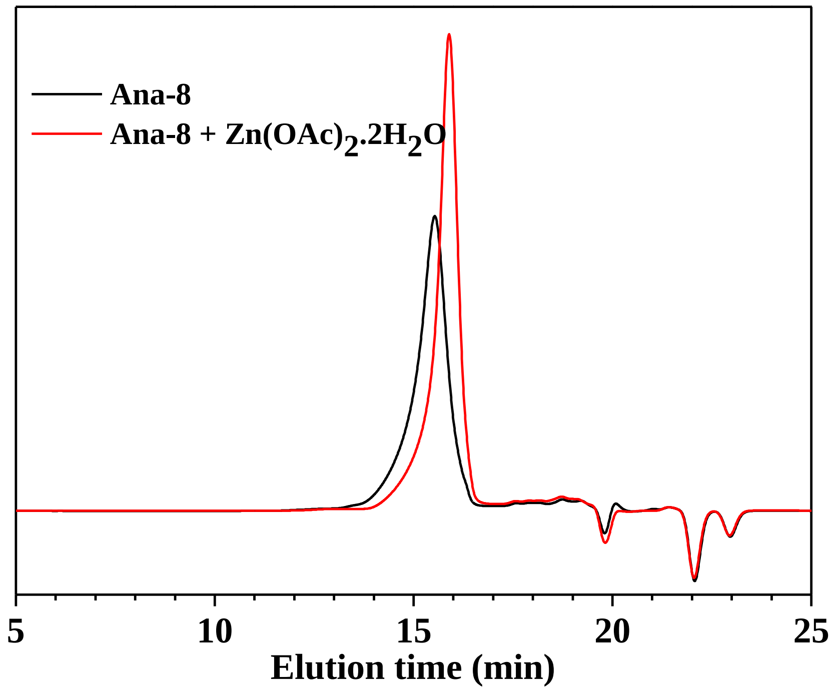

Figure S11. GPC curves of PBLG with Ana-**8** with or without $Zn(OAc)_2 \cdot 2H_2O$. $[Zn(OAc)_2 \cdot 2H_2O]$/[Ana-8]/[BLG-NCA] = 1/1/25, [BLG-NCA] = 0.75 M, at 25 ºC in CH_2Cl_2.

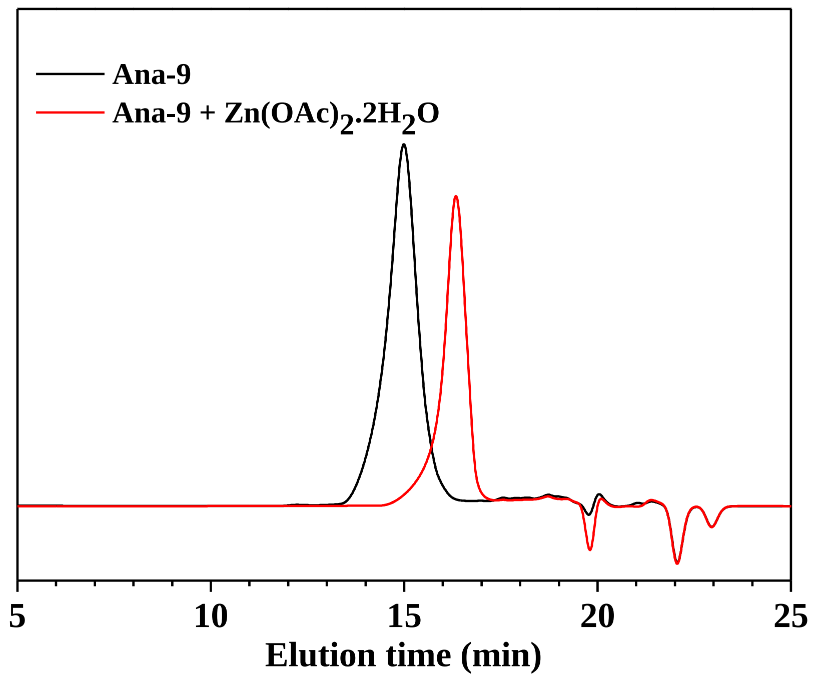

Figure S12. GPC curves of PBLG with Ana-**9** with or without $Zn(OAc)_2 \cdot 2H_2O$. $[Zn(OAc)_2 \cdot 2H_2O]$/[Ana-9]/[BLG-NCA] = 1/1/25, [BLG-NCA] = 0.75 M, at 25 ºC in CH_2Cl_2.

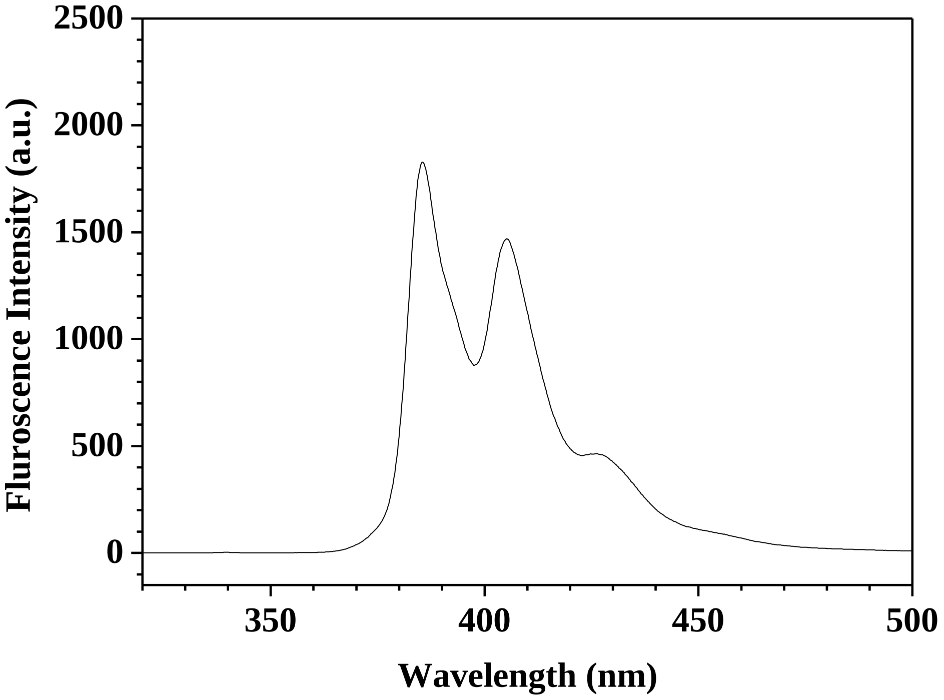

Figure S13. Fluorescent spectrum of PBLG initiated by a combination of $Zn(OAc)_2 \cdot 2H_2O$ with 1-aminopyrene.

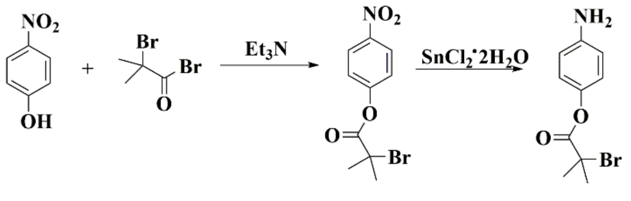

Scheme S1. Synthesis route of Ana-**9**.

The synthesis of Ana-9.

4-Nitrophenol (2.78 g, 0.02 mol), triethylamine (4.04 g, 0.04 mol) and THF 200 mL were placed in one three-neck round bottomed flask. Bromoisobutyryl bromide (4.28 g, 0.02 mol) was added slowly with stirring. After 6 hours, the reaction was filtered and THF was removed in vacuum to obtained 2-bromo-2-methylpropionic acid 4-nitrophenyl ester. The 2-bromo-2-methylpropionic acid 4-nitrophenyl ester (1.44 g, 0.005 mol) and $SnCl_2 \cdot 2H_2O$ (0.025mol) were dissolved in ethyl acetate (100 mL). The mixture was heated under reflux for 1 h at 80 °C, cooled, and made basic (pH 8-9) using 5% sodium bicarbonate aqueous solution. Distilled water (200 mL) was added and the ethyl acetate layer separated. The organic layer was washed with saturated brine solution (3 ×100 mL) followed by distilled water (2 × 100 mL). The organic layer was dried with magnesium sulfate, and the solvent was removed in vacuo. This gave a slightly brown crystalline product **9**.

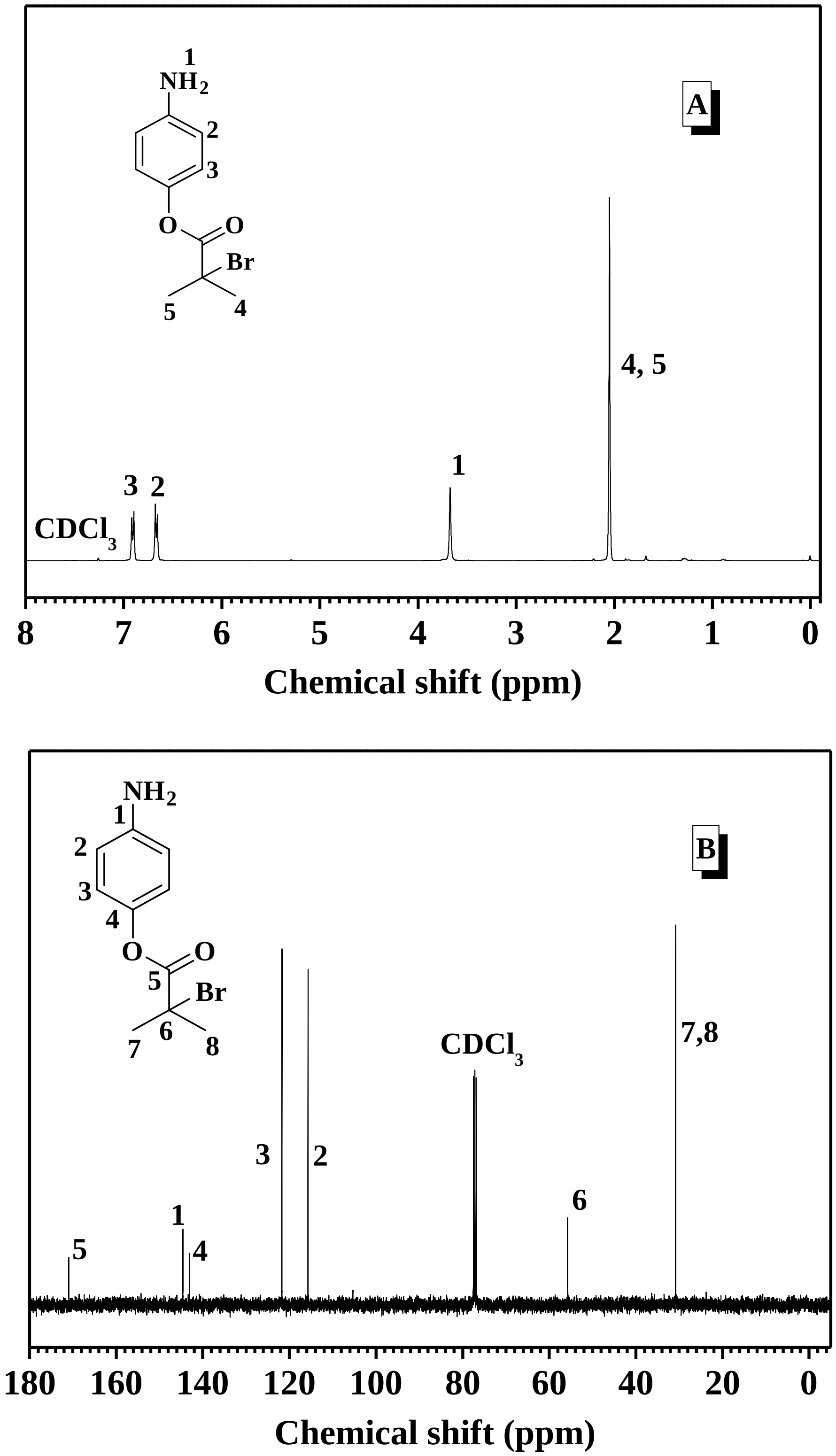

Figure S14. ^{1}H NMR (A) and ^{13}C NMR (B) spectra of 2-bromo-2-methylpropionic acid 4-nitrophenyl ester.

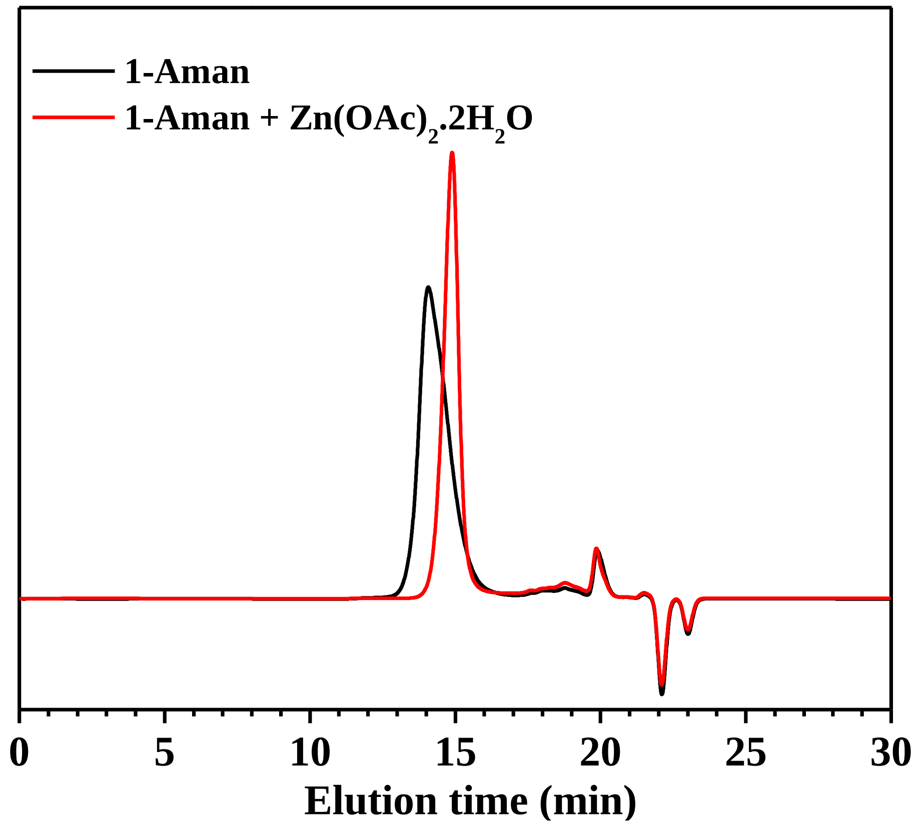

Figure S15. GPC profiles of PBLG initiate by Ana-**10** with or without $Zn(OAc)_2$•$2H_2O$. [BLG-NCA]/[$Zn(OAc)_2$•$2H_2O$]/[Ana-10] = 50/1/1, [BLG-NCA] = 0.75 M, at 25 °C in CH_2Cl_2.

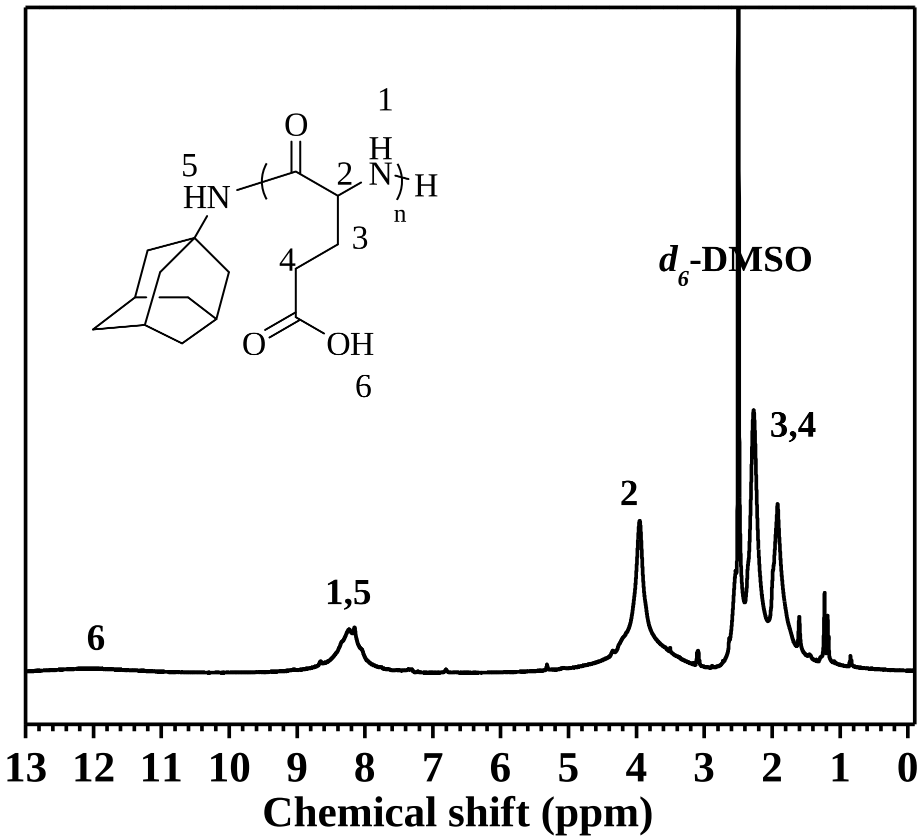

Figure S16. ^{1}H NMR spectrum of Aman-capped PLG.
